# Antifungal Effect of Triglycerol Monolaurate Synthesized by Lipozyme 435-Mediated Esterification

**DOI:** 10.4014/jmb.1910.10043

**Published:** 2020-01-23

**Authors:** Song Zhang, Jian Xiong, Wenyong Lou, Zhengxiang Ning, Denghui Zhang, Jiguo Yang

**Affiliations:** 1 School of Food Science and Engineering, South China University of Technology, 381Wushan Road, Guangzhou 510641, P.R. China; 2 Innovation Center of Bioactive Molecule Development and Application, South China Institute of Collaborative Innovation, Xuefu Road, Dongguan 221116, P.R. China

**Keywords:** Triglycerol monolaurate, enzymatic synthesis, purification, antifungal activity, toxin inhibition

## Abstract

This study was designed to synthesize triglycerol monolaurate (TGML) with Lipozyme 435 as the catalyst, and explore its effects on the growth of *Aspergillus parasiticus* (*A. parasiticus*) and *Aspergillus flavus* (*A. flavus*) and the secretion of aflatoxin b1. The highest content of TGML (49.76%) was obtained at a molar ratio of triglycerol to lauric acid of 1.08, a reaction temperature of 84.93°C, a reaction time of 6 h and an enzyme dosage of 1.32%. After purification by molecular distillation combined with the washes with ethyl acetate and water, the purity of TGML reached 98.3%. Through characterization by electrospray-ionization mass spectrometry, infrared spectrum and nuclear magnetic resonance, the structure of TGML was identified as a linear triglycerol combined with lauroyl at the end. Finally, the inhibitory effects of TGML on the growths of *A. parasiticus* and *A. flavus* and the secretion of aflatoxin b1 were evaluated by measuring the colony diameter, the inhibition rate of mycelial growth and the content of mycotoxin in the media. The results indicated that TGML had a stronger inhibitory effects on colony growth and mycelial development of both toxic molds compared to sodium benzoate and potassium sorbate, and the secretions of toxins from *A. parasiticus* and *A. flavus* were completely suppressed when adding TGML at 10 and 5 mM, respectively. Based on the above results, TGML may be used as a substitute for traditional antifungal agents in the food industry.

## Introduction

A variety of foods and agricultural products, including oil seeds, grains, tree nuts, and dried fruits, are susceptible to contamination by toxin-producing fungi [1]. *Aspergillus*, *Penicillium* and *Fusarium* are common harmful fungi [2]. They not only cause huge economic losses to the food industry and agriculture [3, 4], but also produce some mycotoxins with hepatotoxicity, nephrotoxicity, immunotoxicity, teratogenicity and carcinogenic capacity [5, 6]. Among the studied mycotoxins, the most dangerous is aflatoxin b1, which is the main secondary metabolite of *Aspergillus parasiticus* (*A. parasiticus*) and *Aspergillus flavus* (*A. flavus*) [7]. Due to their heat resistance, aflatoxins on contaminated foods are easily transferred to the diet even if cooked at high temperatures, which poses a great threat to people’s health [8]. Therefore, aflatoxin b1 has been classified as a group 1 carcinogen by the International Agency for Research on Cancer [9].

In order to maintain the safety of foods and agricultural products and extend shelf life, medium-chain fatty acids and their monoglycerides have been widely used to control the growth of hazardous fungi and the secretion of mycotoxins [10-12]. For example, Clelia Altieri reported that 20 ppm of lauric acid completely inhibited the growth of *Fusarium oxysporum* DSMZ 2018 and *Fusarium avenaceum* DSMZ 62151 on potato dextrose agar within 30 days, which was significantly stronger than palmitic acid, myristic acid and their monoglycerides [12]. However, these fatty acid preservatives are generally water insoluble [13], which severely limits their use in food preservation. Currently, although some researchers have proposed the construction of microemulsions [14], liposomes [15] and nanocapsules [16] to improve their solubility, the usage of these formulations in food is challenged by low stability, high cost and complicated preparation process. Therefore, it is necessary to develop a lipid additive with good water solubility as the substitute for fatty acids and their monoglycerides for food preservation.

Triglycerol mono-fatty acid ester is a kind of polyglycerol ester of fatty acids that has excellent emulsion stability [17] and is recognized by the United States and European Union as a safe substance that can be added to foods [18]. In recent years, the inhibitory activities of polyglycerol monoesters against bacteria and yeast have been reported [19, 20]. We also found that triglycerol monolaurate (TGML) has superior antibacterial activity and inhibitory stability against gram-negative bacteria, gram-positive bacteria and yeasts than that of sodium benzoate (SB) and potassium sorbate (PS) [21]. However, there are few reports on the inhibitory effect of polyglycerol mono-fatty acid esters on toxic molds.

Generally, the syntheses of polyglycerol fatty acid esters mainly involves chemical catalysts [22] or lipases [23]. Specifically, use of the chemical method can easily generate by-products and unpleasant flavors, which leads to difficulties in subsequent separation and purification and makes them unsuitable for use as food additives in the food industry [24]. The enzymatic method is a green preparation process with the advantages of mild reaction conditions and fewer by-products, which has attracted the attention of many researchers. Although some polyglycerol fatty acid esters, such as decaglycerol laurate, oligoglycerol linoleate and polyglycerol polyricinoleate, have been synthesized for use as food emulsiﬁers using enzyme catalysts recently [23, 25, 26], the preparation of antimicrobial polyglycerol fatty acid esters is different from that of polyglycerol esters used as emulsifiers. According to previous report [27], the antibacterial active ingredient in fatty acid glycerides is mainly monoglyceride, excluding diglyceride and triester. Similarly, it is speculated that polyglycerol monoester is the main antibacterial active ingredient in polyglycerol fatty acid esters based on recent studies [19-21]. However, few studies have reported enzymatic synthesis and yield optimization of polyglycerol monoester.

As we know, the usual purification methods of polyglycerol fatty acid esters involve column chromatography [17] and distillation method [28]. Although column chromatography can provide a high purity product, it usually requires use of a highly toxic reagent such as chloroform, thus the purified product is not suitable as a food additive. Besides, its purification rate is slow and limited to laboratory preparation. As for the distillation method, it is generally difficult to obtain a product of high purity, which severely limits its application in antibacterial experiment. Therefore, it is necessary to develop a new purification method that is low-toxic, fast, and can produce high-purity products on a large scale.

In this study, TGML was synthesized by triglycerol and lauric acid catalyzed by Lipozyme 435 and optimized by response surface test. Then, the crude ester was purified by molecular distillation in combination with ethyl acetate and water washes. The purified TGML was verified by electrospray ionization mass spectrometry (ESI-MS), infrared spectrum (IR) and nuclear magnetic resonance (NMR). Finally, the inhibitory effects of TGML on the growth of *A. parasiticus* and *A. flavus* and the secretion of aflatoxins b1 were explored with comparison to SB and PS.

## Materials and Methods

### Materials

Glycerol (purity ≥ 99.0%), ethanol (purity ≥ 99.7%), ethyl acetate (purity ≥ 99.5%) and phosphoric acid (purity ≥ 85.0%) were purchased from Tianjin Damao Chemical Reagent Co. (China). Lauric acid (purity ≥ 98.0%), SB (purity ≥ 99.0%), PS (purity ≥ 99.0%), and sodium hydroxide (purity ≥ 98.0%) were obtained from Aladdin Biochemical Technology Company (China). Lipozyme 435 was obtained from Novozymes Co. (Denmark). All water is purified by Mili-Q (Advantage A10, Merck KgaA, Germany).

### Strain Cultures

*A. parasiticus* ATCC 36537 and *A. flavus* ATCC 28539 were purchased from Beina Biotechnology Co. (China). After strain activation, two molds were inoculated on potato dextrose agar (PDA). After cultivation at 28°C for 5-7 days, the fungal spores were collected in sterile water by scraping the mold colonies with sterile inoculation loop and shaking with several glass drops for 5 min followed by filtration with double-layer lens paper. Finally, the absorbance of the spore suspension at 600 nm was adjusted to approximately 0.3, which corresponded to a spore concentration of 1-5× 10^6^ CFU/ml.

### Synthesis and Purification of Triglycerol

The synthesis of triglycerol was carried out according to the method of Bin Peng with some modifications [29]. Glycerol (500 g) and sodium hydroxide (10 g, 2 wt% of glycerol) were added into a 2 L three-necked flask which was heated by a digital magnetic stirring thermostat (KDM, Tianjin Saidlis Experimental Analytical Instrument Manufacturing Co., China). Before heating, nitrogen was passed into the flask to remove air. The reaction is carried out at 260°C for 3 h. After the reaction, the temperature dropped to below 100°C, the reaction product was collected and neutralized with phosphoric acid. Subsequently, 5 volumes of absolute ethanol were added to precipitate the phosphate and then removed by distillation under reduced pressure. Finally, the synthesized triglycerol was purified by two-stage molecular distillation (170°C/20 Pa; 200°C/20 Pa) with a scraping speed of 300 rpm. The fraction between 170 and 200°C was final triglycerol.

The purified triglycerol was analyzed using an HPLC device (Waters e2695, Waters Corp., USA) equipped with a refractive index detector. A Luna NH_2_ column (250 × 4.6 mm, 5 μm) was used with acetonitrile/water (85/15, v/v) as the mobile phase at a flow rate of 1.0 ml/min. The temperatures of column and flow cell were both 30°C. As shown in Fig. S1 (in supplementary file), the purity of final triglycerol was up to 94.2%. The average degree of polymerization of polyglycerol was detected by the method of REN Chun-fang [30]. After purification by molecular distillation, the average degree of polymerization of the final product was 2.90, which confirmed that the purified polyglycerol was triglycerol.

### Enzymatic Synthesis of TGML

Triglycerol, lauric acid and Lipozyme 435 were added to a 1 L three-necked flask, and the reaction was carried out at 300 rpm. In the single factor experiment, the percentage of TGML (PT, %) in crude ester was determined under different molar ratio of triglycerol to lauric acid (2:1-1:2), reaction temperature (60-100°C), reaction time (4-8 h) and enzyme dosage (0.25-2.00%).

After the reaction, the product was centrifuged at 4,000 ×*g* for 10 min to separate it into three layers. The lower layer contained unreacted triglycerol, which could be used as the raw material for the next synthesis. The intermediate layer contained Lipozyme 435, which could be recovered by filtration. The upper layer was mainly crude triglycerol laurate, and the content of TGML could be determined by an HPLC system equipped with an evaporative light-scattering detector (ELSD). The operating parameters of ELSD were as follows: drift tube temperature (80°C), nitrogen flow rate (2.0 L/min), gain (1) and impactor (on). In addition, a Kinetex C18 column (250 × 4.6 mm, 5 μm) was used with acetonitrile/water (60/40, v/v) as the initial mobile phase and gradually changed to 100% acetonitrile after 5 min. The flow rate was 1.0 ml/min with a column temperature of 40°C.

### Response Surface Assay

Based on the results of the single factor experiment, a three-factor, three-level Box-Behnken design was performed to optimize the synthesis of TGML. The factors studied in this assay included the molar ratio of triglycerol to lauric acid, the reaction temperature and the enzyme dosage. The response value was the percentage of TGML in the synthesized triglycerol laurate. The reaction time was set to 6 h for all assays. Design Expert software (version 11) was used to perform variance and regression analysis of the experimental results.

### Purification Method

The synthesized crude ester was firstly purified by a molecular distillation device (DCH-80, Zhengzhou Ruida Grain and Oil Technology Co., China) with a distillation temperature of 170°C, a vacuum degree of 20 pa and a scraping speed of 300 rpm, and the heavy phase was collected. Then the collected fraction was again treated by molecular distillation at a higher distillation temperature (200°C) with other parameters unchanged. The light phase was harvested at this stage.

Considering that the parameters of molecular distillation of triglycerol and TGML were similar, which implied that the triglycerol monoester purified by molecular distillation may be doped with triglycerol, further purification was required. After the two molecular distillations, the collected light phase was dispersed in 10 volumes of ethyl acetate, and then washed with 5 volumes of pure water. After standing for 1 h, the upper layer liquid was collected before vacuum distillation to remove ethyl acetate. The remaining component is pure TGML.

### Characterization of ESI-MS, I_R_ and NMR

The purified TGML was initially identified by MS equipped with an ESI source. The operating parameters were as follows: the ion-spray voltage was 3,500 V with a capillary temperature of 180°C. Mass spectra with a mass range of 50~3,000 m/z were obtained in positive ion mode. Further, the structure of TGML was characterized by I_R_ according to a previous report [17]. The test range was fixed at 187,500~9,250 px^-1^ with a resolution of 4 px^-1^. The ratio of signal to noise was 55,000:1 (peak-to-peak). In addition, the structure of TGML was further determined by ^13^C NMR spectrum. The sample was dissolved in deuterated chloroform, and tetramethylsilane was used as the internal standard with a chemical shift value of 0.

### Inhibition of Colony Growth by TGML

The inhibitory effect of TGML on the colony growth of *A. parasiticus* and *A. flavus* on solid media was studied according to the report by Maristela Martins with some modifications [31]. TGML was added into the PDA media at the following concentrations: 1.25, 2.5, 5, and 10 mM. The negative control contained no antibacterial agent, while the positive controls contained added SB and PS at concentrations of 1.25, 2.5, 5, and 10 mM, respectively. Subsequently, 3 pieces of sterile filter paper (6 mm in diameter) were evenly placed on each solid PDA plate and immediately inoculated with 2 μl of spore suspension (10^6^ CFU/ml). After cultivation at 28°C for 2 days, the diameters of the mold colonies on PDA plates were determined in millimeters.

### Effect of TGML on Mycelial Growth

The inhibitory activity of TGML on the mycelial growth of *A. parasiticus* and *A. flavus* in liquid media was evaluated according to a previous report with slight modifications [10]. TGML was added to potato dextrose broth (PDB) media to achieve concentrations of 1.25, 2.5, 5, and 10 mM, respectively. The media contained SB and PS at 1.25, 2.5, 5, and 10 mM were used as the positive controls, and the medium without an antibacterial substance was regarded as the negative control. Then, 100 μl of the spore suspension (10^6^ CFU/ml) was added to 9.9 ml of the PDB media, and cultured at 28°C for 2 days with 120 rpm shaking. Finally, the mycelia in PDB was filtered and dried to constant weight. The inhibition rate (I_R_, %) of TGML on mycelial growth was calculated as follows:


(1) $$I_{R}(\%)=(\mathrm{Wc}–\mathrm{Ws})⁄\mathrm{Wc}\times 100\%$$

where *Ws* represented the dry weight of mycelia from the samples and positive controls, and *Wc* represented the dry weight of mycelia from the negative control.

### Interference of TGML on Mycotoxin Secretion

The negative controls, positive controls and experimental groups were firstly prepared as described in 2.9. After culturing at 28°C for 14 days, the media were filtered and the filtrate was collected. Subsequently, according to the method of Shao, S. [32], the mycotoxin in the filtrate was extracted twice with dichloromethane before the solvent naturally evaporated in a fume hood. The content of aflatoxin b1 was determined by a fluorescence immunoassay analyzer (Guangzhou Yueyang Biotechnology Co., China). The final toxin content was expressed as μg/ml.

### Statistical Analysis

The data were expressed as the averages ± standard derivation (SD) of three determinations. The statistical comparison was performed by one-way ANOVA followed by Dunnett’s multiple comparisons test in GraphPad Prism 6.00. Different letters were used to represent the significant differences in statistics when *p* ≤ 0.05.

## Results and Discussion

### Effect of Reaction Conditions on the Production of TGML

**Fig. 1:**
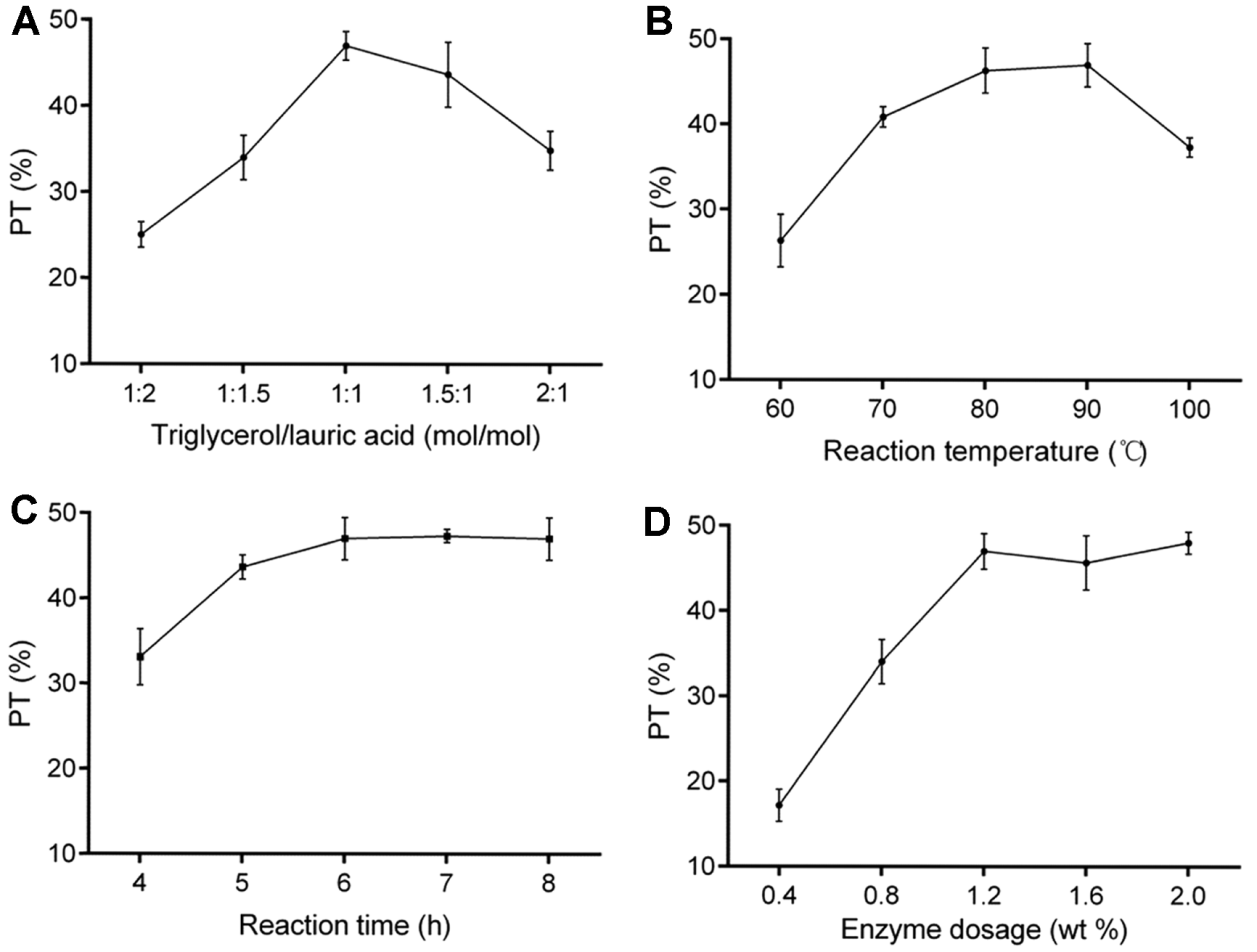
Single factor tests of enzymatic catalyzed esterification of triglycerol and lauric acid. Percentage of TGML (PT, averages ± SD, *n* = 3) at different (**A**) molar ratios of triglycerol to lauric acid (reaction temperature, 80°C; reaction time, 6 h; enzyme dosage, 1.2%), (**B**) reaction temperatures (molar ratio of triglycerol to lauric acid, 1:1; reaction time, 6 h; enzyme dosage, 1.2%), (**C**) reaction times (molar ratio of triglycerol to lauric acid, 1:1; reaction temperature 80°C; enzyme dosage, 1.2%) and (**D**) enzyme dosages (molar ratio of triglycerol to lauric acid, 1:1; reaction temperature 80°C; reaction time, 6 h).

The influence of molar ratio of triglycerol to lauric acid, reaction temperature, reaction time and enzyme dosage on the production of TGML was shown in Figure 1. The content of TGML increased obviously with increase of the molar ratio of triglycerol/lauric acid from 1:2 to 1:1, and the highest content of TGML (47.20 ± 1.66%) occurred at 1:1 (Fig. 1A). When the molar ratio exceeded 1:1, the content of TGML declined rapidly, probably because the remaining triglycerol was not esterified. A similar effect could also be seen in Figure 1B. The content of TGML gradually increased with an increase in reaction temperature from 60 to 90°C, and the maximum PT (46.97 ± 2.56%) was acquired at the temperature of 90°C. However, further increasing temperature to 100°C would result in a decrease in the yield of target product, which may be related to the loss of lipase activity and the decrease of its affinity for the substrate under high temperature conditions [33].

As shown in Fig. 1C, the content of TGML significantly increased as the reaction time increased from 4 to 6 h, and then reached a stable phase in the range of 6-8 h. PT reached its maximum (46.83 ± 2.47%) at 6 h. As for Fig. 1D, it was observed that PT increased from 17.17 ± 1.88 to 47.40 ± 2.10% with increase of the enzyme dosage from 0.4 to 1.2%, and then remained unchanged at the dose range of 1.2 to 2.0%. The highest content of TGML (47.40 ± 2.10%) appeared at the lipase dosage of 1.2%.

Similar studies on the effect of reaction conditions on the synthesis of polyglycerol fatty acid esters by single factor experiment have also been reported. For example, Bin Peng studied the esterification synthesis of polyglycerol and rice-bran oil, C. camphora seed oil, or acetic acid catalyzed by Lipozyme 435, and found that the best esterification efficiency appeared in the substrate molar ratio of 1.5:1 [29]. In addition, in another lipase-catalyzed transesterification of decylglycerol and methyl laurate, the highest conversion of methyl laurate was observed at a substrate molar ratio of 2:1, a reaction temperature of 65°C, and an enzyme concentration of 7% [23]. The difference reported by the above literature may be because of the different reaction substrates, esterification methods and detection indicators.

### Model Fitting and Response Surface Optimization

The response surface design (Box-Behnken) of the esteriﬁcation of TGML was displayed in Table 1. After fitting with the intercept model, the F-value and R^2^ in ANOVA analysis were 227.81 (at *p* < 0.0001) and 0.9966, respectively, indicating that this fitting could correctly reflect the interactions among the three factors (Table 2). The lack of fit test was not significant (F-value of 0.068 at *p* > 0.05), which further showed that the model fitted the experimental results well. The high fitting coefficient (R^2^ of 0.9966, adjusted R^2^ of 0.9922) suggested that the model could accurately reflect the trend of experimental data. Based on the F value corresponding to each factor, an order of importance of three factors was obtained as follows:


(2) $$\mathrm{PT}(\%)=46.72-3.87A+2.72B+6.44C+0.09\mathrm{AB}-0.20\mathrm{AC}+0.67\mathrm{BC}-{7.29A}^{2}-{3.31B}^{2}-{6.22C}^{2}(2)$$

enzyme dosage > substrate molar ratio > reaction temperature. The second-order fitting formula for PT calculation was shown as the following: where A was the molar ratio of triglycerol to lauric acid, B was the reaction temperature, and C was the enzyme dosage.

**Fig. 2:**
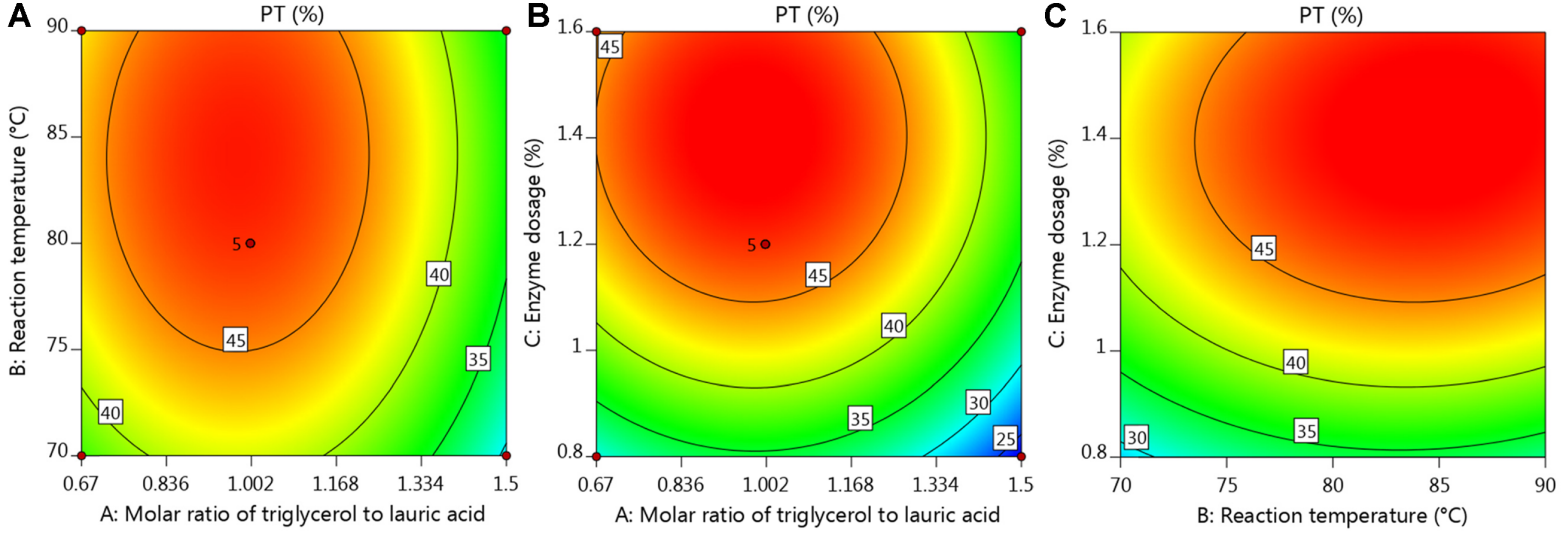
Contour plots of the interactions among the molar ratio of triglycerol to lauric acid, reaction temperature and enzyme dosage. PT as a function of (**A**) triglycerol/lauric acid molar ratio and reaction temperature with the enzyme dosage set to 1.2%, (**B**) triglycerol/lauric acid molar ratio and enzyme dosage with the reaction temperature set to 80°C, and (**C**) reaction temperature and enzyme dosage with the triglycerol/lauric acid molar ratio set to 1.00.

The influence of the interaction between substrate molar ratio, reaction temperature and enzyme dosage on PT was recorded in Fig. 2. PT was influenced in the following order: BC > AC > AB. The optimized conditions for TGML synthesis calculated by Design Expert software were the triglycerol/lauric acid molar ratio of 1.08, the reaction temperature of 84.93°C, the reaction time of 6 h and the enzyme dosage of 1.32%. The predicted PT was up to 49.76%. Small differences occurred between the optimized conditions and the actual optimal parameters, which may be derived from the interaction among the three factors. Further, we synthesized TGML again under the optimal conditions, and the PT was 49.34%, which proved the accuracy and reliability of the fitted model.

Recently, many studies have also reported the optimization of the synthesis of polyglycerol fatty acid esters. For instance, Bin Peng obtained the best parameters (reaction temperature of 85°C, reaction time of 6 h, enzyme dosage of 1.4% and substrate molar ratio of 1.35) for esterification of polyglycerol and rice-bran oil through a three-factor, three-level Box-Behnken test, and the predicted esterification efficiency was 69.82% [29]. Similarly, in another report, the esterification of oligoglycerol with linoleic acid was also optimized by Box-Behnken assay, and the maximum esterification efficiency (96.15%) occurred in the reaction time of 4.52 h, the reaction temperature of 90°C, the enzyme dosage of 2%, the molar ratio of oligoglycerol to linoleic acid of 1.59:1 and no water addition [26]. In addition, an orthogonal test was also used to optimize the synthesis of decaglycerol laurates, and the highest conversion of methyl laurate reached 83.3 ± 1.5% under optimal conditions (reaction time 4.5 h, rotating speed 180 rpm, enzyme dosage 8%, and water content 5%) [23]. Compared to the above reports, the response value in this study was lower, probably because the optimization object of this study is a component in the final product.

### Purification of TGML

**Fig. 3:**
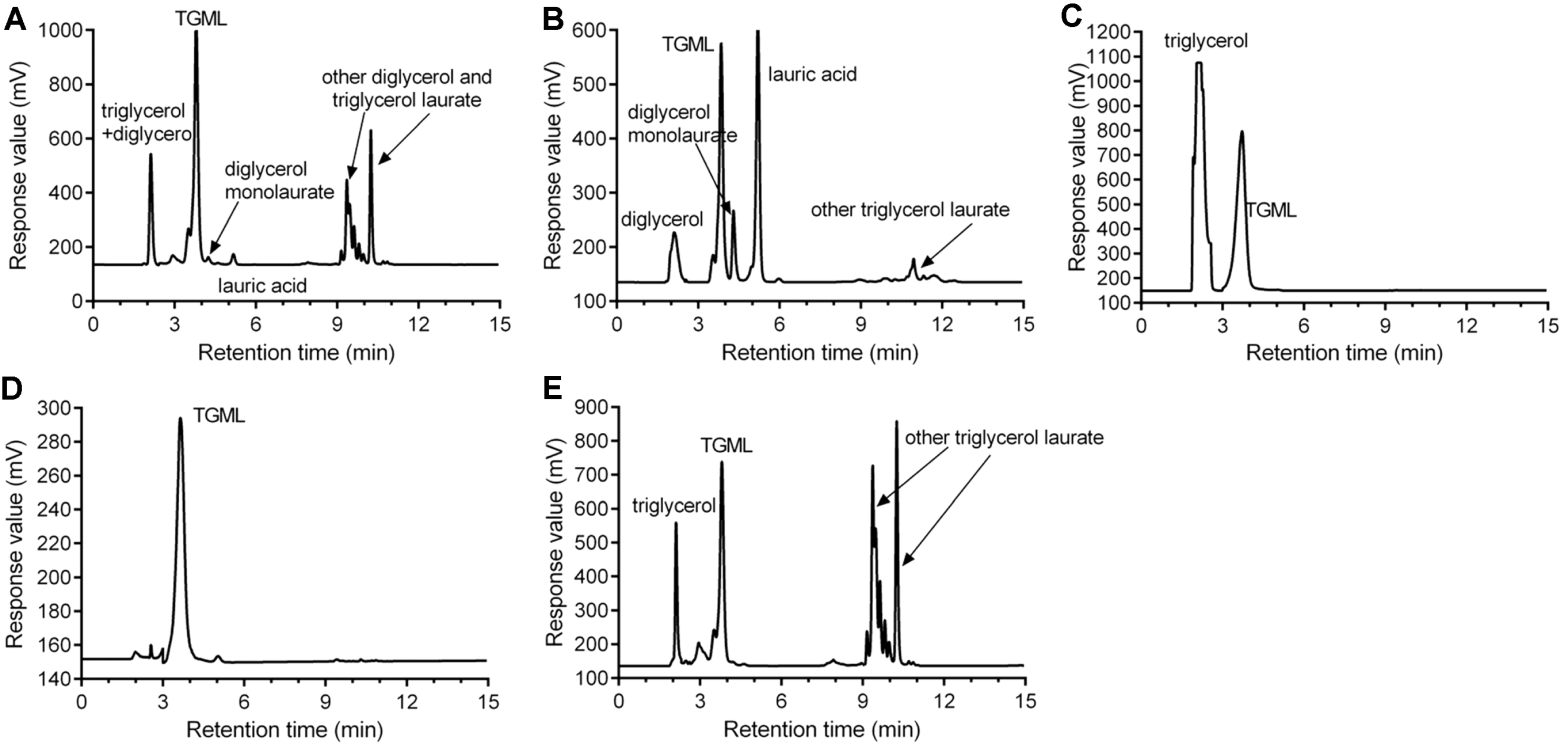
HPLC chromatograms of synthetic crude ester (A) and the light phase (B), intermediate phase (C), intermediate phase followed by ethyl acetate and water wash (D) and heavy phase (E) separated by molecular distillation at 170-220°C.

The synthesized triglycerol laurate was purified by molecular distillation combined with ethyl acetate and water washes (Fig. 3). Based on the determination of HPLC-ELSD, the crude ester contained di- and tri-glycerol (9.58%), TGML (47.34%), diglycerol monolaurate (1.31%), lauric acid (2.70%) and other diglycerol and triglycerol laurate (36.22%) (Fig. 3A). After purification by two-stage molecular distillation (170°C/20 pa, 200°C/20 pa), the middle-phase fraction contained only triglycerol and TGML (Fig. 3C). Subsequently, the triglycerol in the ester was removed by the extraction of TGML by ethyl acetate and the dissolution of triglycerol by water, and the purity of final TGML was up to 98.3% (Fig. 3D).

Currently, column chromatography and distillation technique are often used in the purification of polyglycerol fatty acid esters. For instance, TGML crude ester was purified to 98.7% through a silica gel column using chloroform/methanol (90:10) as the eluting solvent [17]. Although the reported purity was very similar to the results in this study, the disadvantages are obvious, such as the use of chloroform (highly toxic) and very slow purification rate. In addition, J. Holstborg reported that diglycerol monoester could be purified to more than 80% by laboratory distillation process [28]. Obviously, its purity could not meet the requirements of many scientific assays, including antibacterial test.

### Characterization of ESI-MS, I_R_ and NMR

The composition of purified TGML characterized by ESI-MS was shown in Table 3. The purified TGML contained [lauric acid + monoester of triglycerol + Na]^+^ (1H) (100%), [lauric acid + monoester of triglycerol + Na]^+^ (2H) (19.9%), [lauric acid + monoester of triglycerol + Na]^+^ (3H) (2.9%), [lauric acid + monoester of tetraglycerol + Na − H_2_O]^+^ (2.0%), [lauric acid + diester of triglycerol + Na]^+^ (0.8%) and [lauric acid + triester of tetraglycerol + Na − H_2_O]^+^ (1.1%). As the dehydration reaction did not occur during the electrospray ionization process [34], it was speculated that the removal of water molecules in the above positive ions might be due to etherification of the adjacent hydroxyl groups in polyglycerol. Based on the ion intensity, the TGML containing three hydrogen isotopes was the most abundant, which was consistent with the result of purified TGML analyzed by HPLC-ELSD.

The functional groups of TGML determined by I_R_ were shown in Fig. S2 (in supplementary file). C=O (1,738 cm^-1^), C-O-C(symmetrical and asymmetrical stretching vibration at 1,045 and 1,116 cm^-1^) and OH (3,378 cm^-1^) illustrated the presence of an ester bond, an ether bond and a hydroxyl group in TGML, respectively. This was in agreement with the results of HPLC-ELSD assay.

In addition, the results of ^13^C NMR spectra of TGML were shown in Fig. S3 (in supplementary file). It was found that the signals on the triglycerol carbon skeleton were located at 60-72 ppm, and no signal exceeded 80 ppm. Therefore, based on the previous reports [34, 35], the triglycerol skeleton was judged as linear triglycerol. Besides, referring to the chemical shifts of terminal carbon (62.92 ppm) and intermediate carbon (70.83 ppm) of polyglycerol reported by Fenlong Wan [35], the chemical shifts of terminal and intermediate carbons on the polyglycerol backbone of TGML in this study were 64.00, 62.63 ppm and 67.55, 68.69, 69.90 ppm, respectively. The downfield chemical shift showed an occurrence of typical acylation reaction, indicating that lauric acid was mainly bound to the terminal hydroxyl group of triglycerol in enzymatic esterification, which could be explained by the higher reactive activity of primary hydroxyl groups than secondary hydroxyl groups due to the space hindrance effect [36]. Therefore, we concluded that the structure of purified TGML was mainly linear triglycerol combined with lauroyl at the end.

### Inhibition of TGML on Colony and Mycelial Growths of Molds

**Fig. 4:**
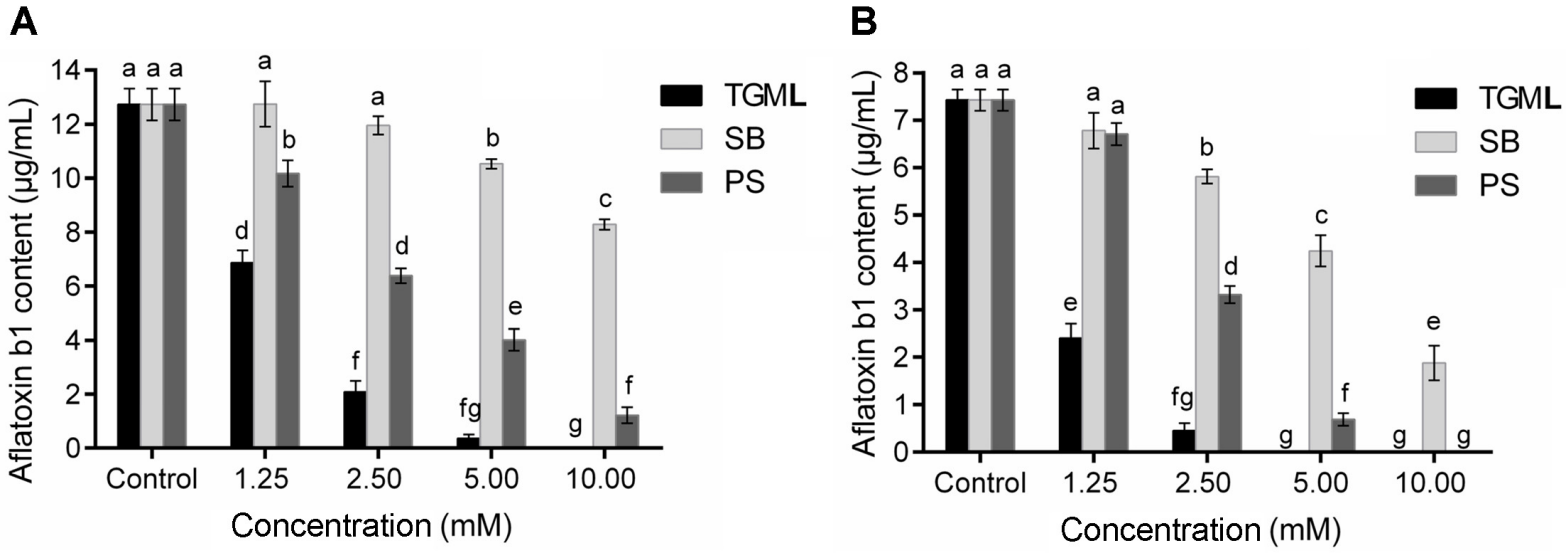
Content of aflatoxin b1 produced by *A. parasiticus* (A) and *A. flavus* (B) treated with TGML, SB and PS at 1.25, 2.5, 5 and 10 mM. Columns not having the same letters are considered significantly different (*p* ≤ 0.05).

The inhibition of TGML on the growth of *A. parasiticus* and *A. flavus* colonies was clearly seen in Fig. 4. After treatment with TGML at 1.25, 2.5, and 5 mM, the colony areas of both molds were significantly reduced, even less than the same concentrations of SB and PS. Further, the antifungal effect of TGML was quantified by measuring the diameters of two *Aspergillus* colonies. As shown in Table 4, compared to the control (25.60 ± 0.60 mm), the diameters of the colonies of *A. parasiticus* dropped significantly to 21.00 ± 0.20, 17.87 ± 0.61, and 15.20 ± 0.20 mm in the treatment of TGML at 1.25, 2.5, and 5 mM, respectively, smaller than the colony sizes treated by the same concentrations of SB and PS . As for *A. flavus*, the inhibitory effect of TGML was more obvious. After adding TGML into the solid media at 1.25, 2.5, and 5 mM, the colony diameters of *A. flavus* would decrease by 8.53, 11.20, and 13.66 mm, respectively, which was also superior to the inhibitory effects of SB and PS.

In addition, the effects of TGML on the I_R_ of mycelial growths of two toxic molds in liquid media were shown in Table 5. As the concentration of TGML increased from 1.25 to 10 mM, its I_R_ on the mycelial growth of *A. parasiticus* increased from 12.57 ± 0.41 to 91.58 ± 1.03%. In other words, the mycelial growth of toxic mold was almost totally inhibited at 10 mM. However, the maximum I_R_ values of SB and PS on the mycelial growth of this mold were only 56.43 ± 1.24% and 62.57 ± 0.21% in the concentration range of 1.25 to 10 mM. In the test of *A. flavus*, compared to the 1.25, 2.5, 5, and 10 mM of SB and PS, the I_R_ of mycelial growth by the same concentrations of TGML increased by 10.37%, 18.91%, 26.65%, 42.81%, and 8.87%, 16.88%, 21.09%, 40.13%, respectively. Besides, it was found that the inhibitory effect of TGML on *A. parasiticus* in liquid media was close to that of *A. flavus* due to their similar I_R_ values.

From the above results, it was concluded that the inhibitory activity of TGML against *A. parasiticus* and *A. flavus* in the media was obviously stronger than that of SB and PS, which might be related to the effects on the formation of hyphae or pseudohyphae [19]. In addition, its inhibitory effect on toxigenic fungi was concentration dependent, which was similar to the results reported by the recent antifungal studies [10-12, 19, 20]. For example, the growths of *Saccharomyces cerevisiae*, *Aspergillus niger* and *Penicillium glaucum* were completely inhibited by monolaurin at 0.32, 0.32, and 0.16 mg/ml, respectively, while the cells or spores of the three fungi were totally removed only when the concentration of monolaurin increased to 1.25, 2.5, and 0.63 mg/ml, respectively [11], indicating that the antifungal effect of monolaurin was directly related to its concentration. Besides, Chikako Ikegawa found that the cell numbers of *Saccharomyces cerevisiae* decreased by 0, 2.2, and 4.3 log CFU/ml, respectively, when incubated with diglycerol monolaurate at 31.3, 62.5, and 125 μg/ml [20], suggesting that diglycerol monolaurate also exerted a lethal effect against yeast cells by a concentration-dependent manner.

### Interference of TGML on Mycotoxin Secretion

The effect of TGML on the production of aflatoxin b1 produced by two toxic molds was shown in Fig. 4. In the test of *A. parasiticus*, when cultured with TGML at 1.25, 2.5, 5, and 10 mM, the amount of aflatoxin b1 secreted by *A. parasiticus* would decrease from 12.73 ± 0.59 μg/ml (in the control) to 6.86 ± 0.46, 2.08 ± 0.41, 0.36 ± 0.15, and 0.00 μg/ml, respectively, lower than the toxin content treated by SB and PS at the same concentrations (Fig. 5A). Similar trends were also observed in the inhibitory assay of *A. flavus* (Fig. 5B). The content of aflatoxin b1 declined rapidly to 0 as the concentration of TGML increased from 1.25 to 5 mM. Compared to SB and PS at the same concentrations, the inhibitory ability of TGML on the secretion of toxin from *A. flavus* was obviously stronger. Besides, it was found that TGML inhibited the secretion of toxin from *A. flavus* stronger than that from *A. parasiticus*, which might be related to the species differences between the two molds.

Recently, many studies on the inhibition of mycotoxin secretion by esters have been reported [1, 6, 37, 38]. Tiago M. Nazareth reported that the content of aflatoxin would decrease by 19.9, 45.29, and 100%, respectively, when incubated with allyl isothiocyanate at 0.1, 1, and 10 μl/l [1]. It was speculated that the inhibition of mycotoxin secretion might be due to the fact that the changes in the physiological environment of fungi affected the signal transduction of toxin biosynthesis [39]. Of course, the specific inhibitory mechanism still needed further exploration.

In conclusion, TGML was synthesized by esterification of triglycerol and lauric acid catalyzed by Lipozyme 435. The highest TGML content (49.76%) was obtained under the following conditions: triglycerol/lauric acid molar ratio (1.08), reaction temperature (84.93°C), reaction time (6 h) and enzyme dosage (1.32%). It was purified to 98.3% by molecular distillation in combination with the washes of ethyl acetate and water. Its structure was proved to be linear triglycerol combined with lauroyl at the end by ESI-MS, I_R_ and NMR. Besides, compared to SB and PS, TGML had a better inhibitory effects on the growth and toxin secretion of *A. flavus* and *A. parasiticus*. Therefore, TGML may be used as a substitute for traditional antifungal agents in the food industry.

## Supplementary material

Supplementary data for this paper are available on-line only at http://jmb.or.kr.



## Figures and Tables

**Table 1:** A three-factor and three-level response surface experiment design (Box−Behnken) of TGML synthesis.

Experiment numbers	Factors			PT (%)

	Molar ratio (mol/mol)	Reaction temperature (°C)	Enzyme dosage (%)	
1	1:1.5	80	0.8	30.49
2	1.5:1	80	1.6	35.63
3	1:1	90	1.6	47.92
4	1.5:1	80	0.8	24.52
5	1:1.5	90	1.2	42.67
6	1:1	90	0.8	33.16
7	1:1	80	1.2	46.27
8	1:1	70	0.8	28.78
9	1.5:1	70	1.2	29.34
10	1:1	70	1.6	40.86
11	1:1	80	1.2	47.13
12	1:1	80	1.2	48.29
13	1:1	80	1.2	47.58
14	1:1.5	70	1.2	37.80
15	1.5:1	90	1.2	34.69
16	1:1.5	80	1.6	43.21
17	1:1	80	1.2	46.75

**Table 2:** ANOVA analysis for quadratic model.

Source	Sum of squares	df	Mean square	F-value	*p*-value	
Model	1024.88	9	113.88	227.81	<0.0001	Significant
A-Molar ratio of triglycerol to lauric acid	120.05	1	120.05	240.16	<0.0001	
B-Reaction temperature	57.84	1	57.84	115.72	<0.0001	
C-Enzyme dosage	324.94	1	324.94	650.05	<0.0001	
AB	0.0309	1	0.0309	0.0618	0.8109	
AC	0.1630	1	0.1630	0.3260	0.5859	
BC	1.80	1	1.80	3.59	0.0999	
A^2^	200.70	1	200.70	401.50	<0.0001	
B^2^	46.01	1	46.01	92.05	<0.0001	
C^2^	162.81	1	162.81	325.70	<0.0001	
Residual	3.50	7	0.4999			
Lack of fit	1.09	3	0.3648	0.6068	0.6448	Not significant
Pure error	2.40	4	0.6012			
Cor total	1028.38	16				
R^2^	0.9966					
Adjusted R^2^	0.9922					

**Table 3:** ESI-MS analysis of purified TGML.

No.	m/z	Component	Intensity (%)
1	445.2782	[lauric acid+ monoester of triglycerol+Na]^+^(1H)	100
2	446.2818	[lauric acid+ monoester of triglycerol+Na]^+^(2H)	19.9
3	447.2827	[lauric acid+ monoester of triglycerol+Na]^+^ (3H)	2.9
4	501.3044	[lauric acid+ monoester of tetraglycerol+Na?H_2_O]^+^	2.0
5	627.4450	[lauric acid+ diester of triglycerol+Na]^+^	0.8
6	867.5663	[lauric acid+ triester of tetraglycerol+Na?H_2_O]^+^	1.1

**Table 4:** Colony diameters of A. parasiticus and A. flavus grown on PDA supplemented with TGML, SB, and PS at 1.25, 2.5 and 5 mM.

	Colony diameter (mm)	

	*A. parasiticus*	*A. flavus*
Control	25.60 ± 0.60 a	25.33 ± 0.31 a
TGML 1.25 mM	21.00 ± 0.20 b	16.80 ± 0.20 c
TGML 2.5 mM	17.87 ± 0.61 c	14.13 ± 0.42 d
TGML 5 mM	15.20 ± 0.20 d	11.67 ± 0.31 e
SB 1.25 mM	25.60 ± 0.87 a	25.93 ± 0.70 a
SB 2.5 mM	24.27 ± 0.50 a	24.80 ± 0.20 a
SB 5 mM	22.53 ± 0.42 b	23.13 ± 0.31 ab
PS 1.25 mM	25.13 ± 0.76 a	22.33 ± 0.50 b
PS 2.5 mM	22.60 ± 0.72 b	20.13 ± 0.42 bc
PS 5 mM	19.33 ± 0.58 c	16.27 ± 0.42 d

The mean values that are not followed by the same letter are significantly different (*p* ≤ 0.05).

**Table 5:** Inhibition rates (%) of mycelial growth of *A. parasiticus* and *A. flavus* grown in PDB supplemented with TGML, SB and PS at 1.25, 2.5, 5, and 10 mM.

	Inhibition rate of mycelial growth (%)	

	*A. parasiticus*	*A. flavus*
Control	0	0
TGML 1.25 mM	12.57 ± 0.41 a	19.34 ± 2.04 ab
TGML 2.5 mM	27.78 ± 0.41 b	33.12 ± 1.36 bc
TGML 5 mM	57.89 ± 2.48 d	51.22 ± 2.42 cd
TGML 10 mM	91.58 ± 1.03 e	85.22 ± 0.45 e
SB 1.25 mM	3.51 ± 1.24 a	8.97 ± 1.21 a
SB 2.5 mM	17.11 ± 2.79 ab	14.21 ± 1.89 a
SB 5 mM	43.13 ± 0.93 c	24.57 ± 1.21 ab
SB 10 mM	56.43 ± 1.24 d	42.41 ± 1.44 c
PS 1.25 mM	6.58 ± 0.93 a	10.47 ± 1.36 a
PS 2.5 mM	22.08 ± 2.38 ab	16.24 ± 1.66 ab
PS 5 mM	46.35 ± 1.14 cd	30.13 ± 2.12 bc
PS 10 mM	62.57 ± 0.21 d	45.09 ± 2.27 cd

The mean values that are not followed by the same letter are significantly different (*p* ≤ 0.05).
