# Supplementary material for: Antifungal Effect of Triglycerol Monolaurate Synthesized by Lipozyme 435-Mediated Esterification
Source: J Microbiol Biotechnol. 2020 Jan 23;30(4):561–70. doi: 10.4014/jmb.1910.10043 (PMC9728257; doi:10.4014/jmb.1910.10043)
Supplement: Supplementary file 1 [file JMB-30-4-561-supple.pdf]

https://doi.org/10.4014/jmb.1910.10043

Supplementary file

Supplemental Figures

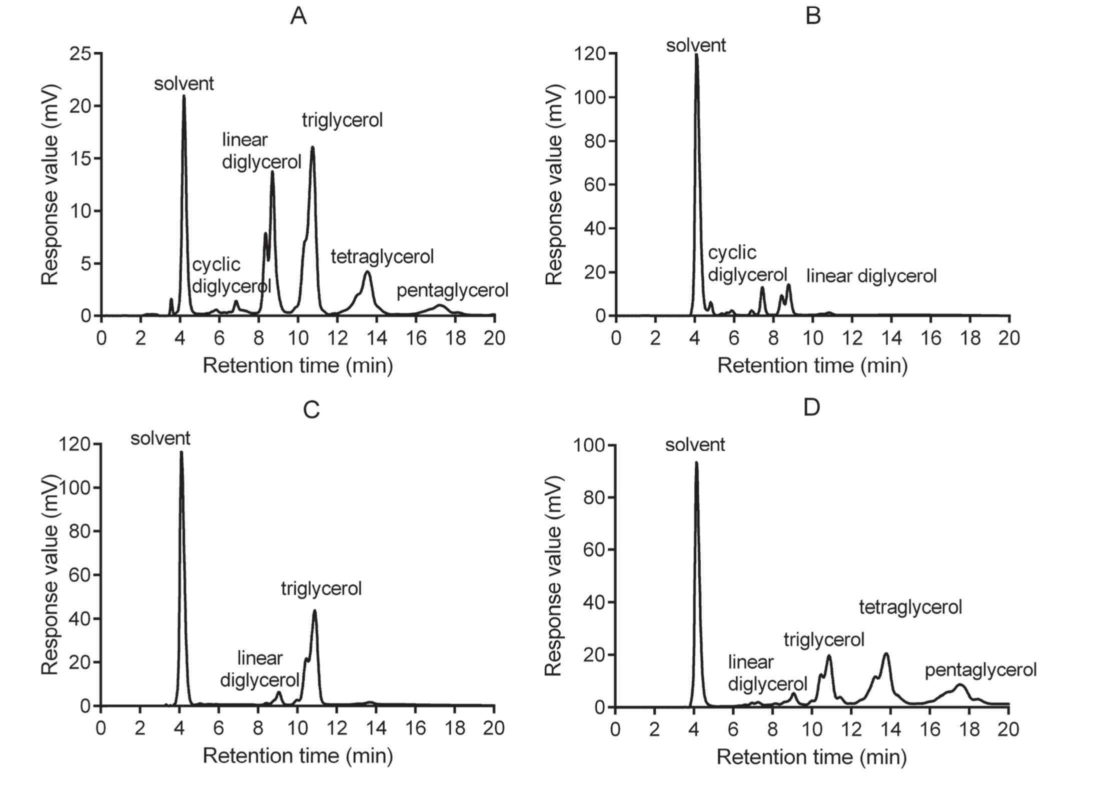

Figure S1 HPLC chromatograms of synthetic crude triglycerol (A) and light phase (B), intermediate phase (C) and heavy phase (D) purified by molecular distillation at 170-200 °C.

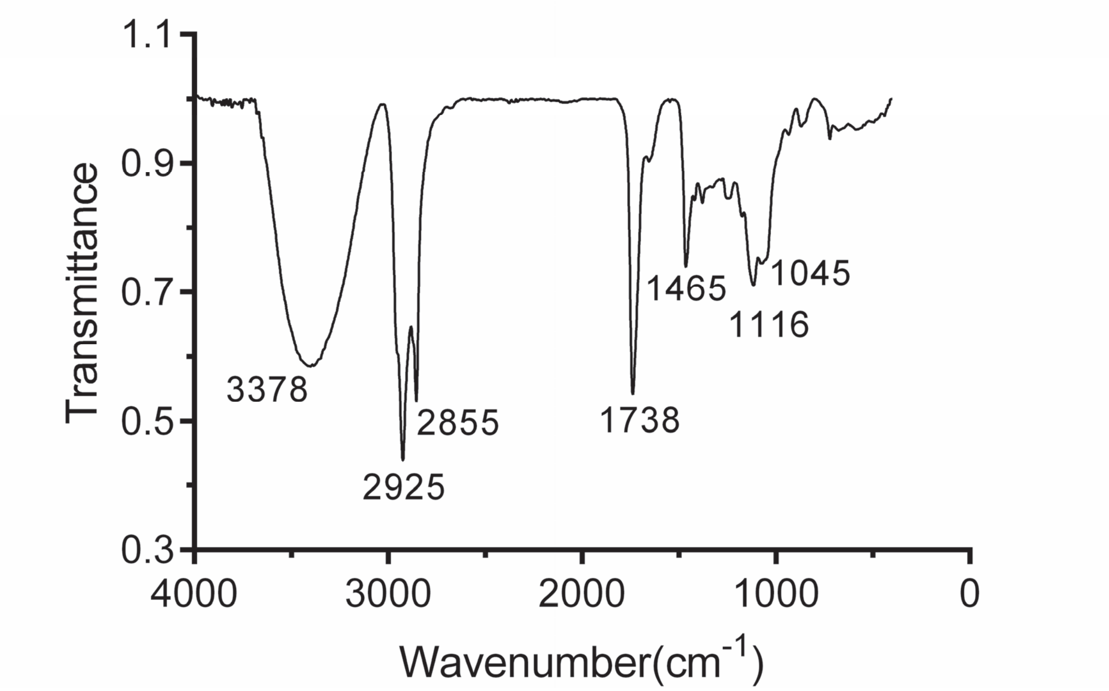

Figure S2 Infrared spectrum of purified TGML.

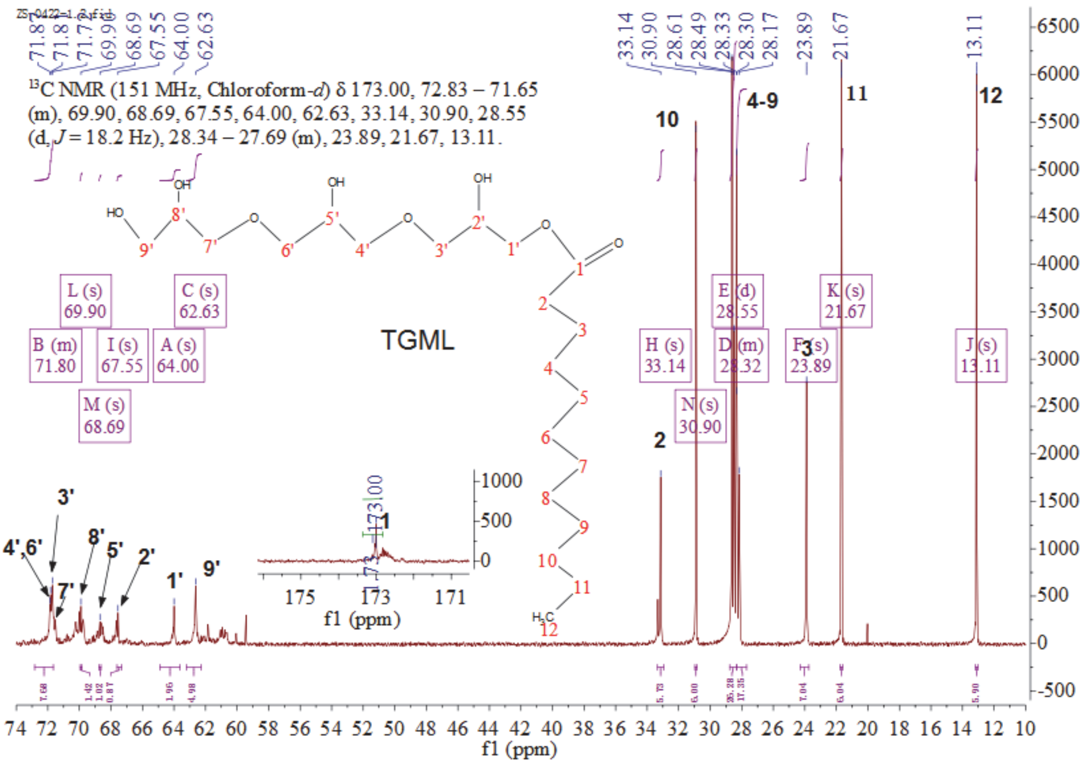

Figure S3 ^{13}C NMR spectrum of purified TGML.

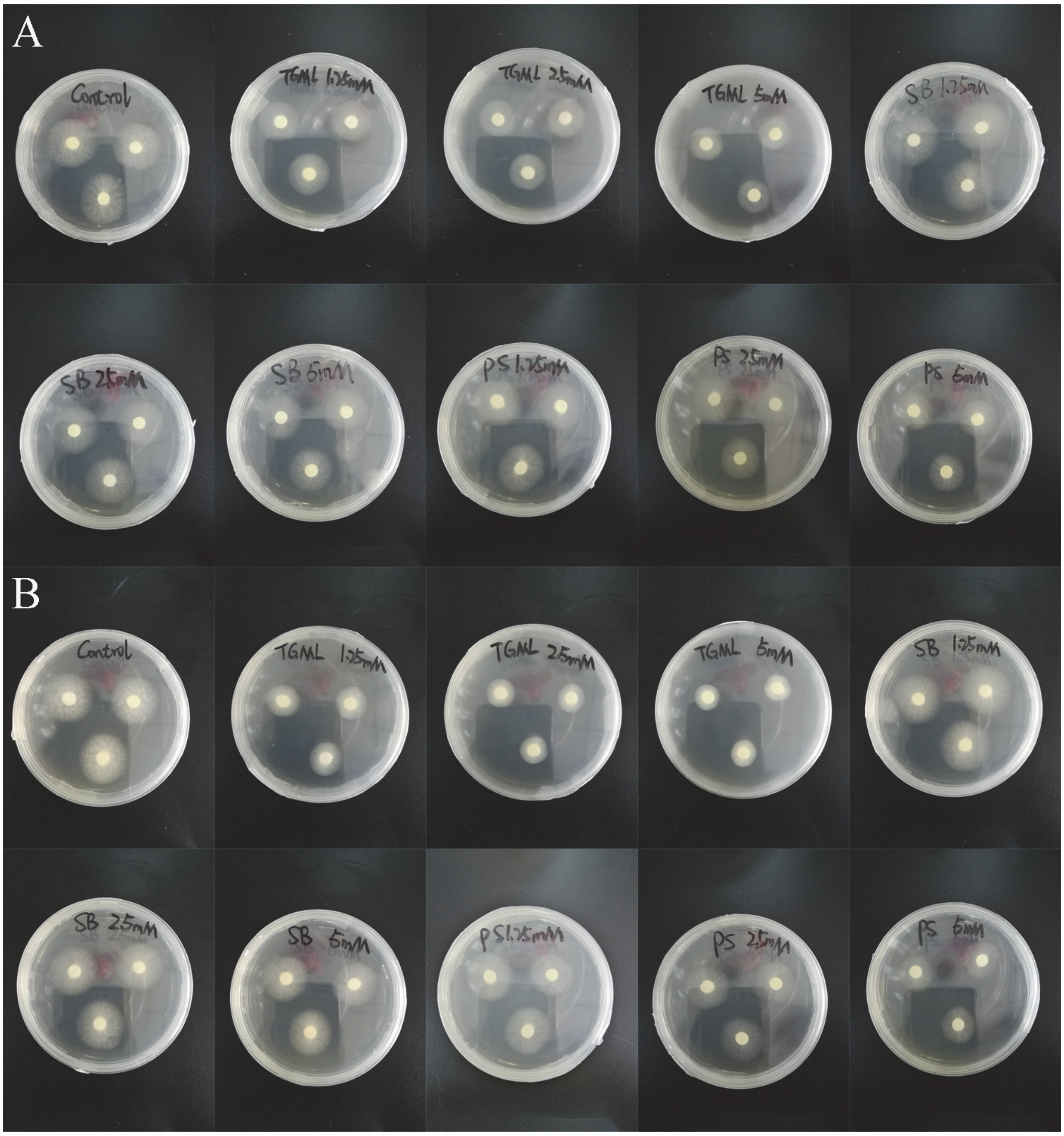

Figure S4 Colony images of *A. parasiticus* (A) and *A. flavus* (B) grown on PDA added with TGML, SB and PS at 1.25, 2.5 and 5 mM.
